# Social Group Size and Shelter Availability Influence Individual Metabolic Traits in a Social Fish

**DOI:** 10.1093/iob/obab032

**Published:** 2021-11-27

**Authors:** Emmanuelle Chrétien, Daniel Boisclair, Steven J Cooke, Shaun S Killen

**Affiliations:** Département de sciences biologiques, Université de Montréal, Campus MIL, 1375 Av. Thérèse-Lavoie-Roux, Montréal, QC H2V 0B3, Canada; Groupe interuniversitaire en limnologie et environnement aquatique (GRIL), Campus MIL, 1375 Av. Thérèse-Lavoie-Roux, Montréal, QC H2V 0B3, Canada; Département de sciences biologiques, Université de Montréal, Campus MIL, 1375 Av. Thérèse-Lavoie-Roux, Montréal, QC H2V 0B3, Canada; Groupe interuniversitaire en limnologie et environnement aquatique (GRIL), Campus MIL, 1375 Av. Thérèse-Lavoie-Roux, Montréal, QC H2V 0B3, Canada; Fish Ecology and Conservation Physiology Laboratory, Department of Biology and Institute of Environmental and Interdisciplinary Science, Carleton University, 1125 Colonel By Drive, Ottawa, ON K1S 5B6, Canada; Groupe interuniversitaire en limnologie et environnement aquatique (GRIL), Campus MIL, 1375 Av. Thérèse-Lavoie-Roux, Montréal, QC H2V 0B3, Canada; Institute of Biodiversity, Animal Health and Comparative Medicine, College of Medical, Veterinary and Life Sciences, University of Glasgow, Graham Kerr Building, Glasgow G12 8QQ, UK

## Abstract

Group living is widespread among animal species and yields both costs and benefits. Presence of conspecifics can restrict or enhance the expression of individual behavior, and the recent social environment is thought to affect behavioral responses in later contexts, even when individuals are alone. However, little is known about how social group size influences the expression of individual physiological traits, including metabolic rates. There is some evidence that shoaling can reduce fish metabolic rates but this variable may be affected by habitat conditions such as shelter availability via density-dependent processes. We investigated how social group size and shelter availability influence Eurasian minnow (*Phoxinus phoxinus*) metabolic rates estimated by respirometry. Respirometry trials were conducted on fish in isolation before and after they were housed for 3 weeks in a social treatment consisting in a specific group size (*n* = 4 or 8) and shelter availability (presence or absence of plant shelter in the experimental tank). Plant shelter was placed over respirometers for half of the duration of the respirometry trials, allowing estimation of minimum daytime and nighttime metabolic rates in both conditions (in the presence or absence of plant shelter). Standard metabolic rate (SMR), maximum metabolic rate (MMR), and aerobic scope were also estimated over the entire trial. Minimum daytime and nighttime metabolic rates estimated while in presence of plant shelter were lower than when estimated in absence of plant shelter, both before and after individuals were housed in their social treatment. After the social treatment, SMRs were higher for fish that were held in groups of 4 as compared with those of fish held in groups of 8, while MMR showed no difference. Plant shelter availability during the social treatments did not influence SMR or MMR. Our results suggest that social group size may directly influence energy demands of individuals, highlighting the importance of understanding the role of group size on variations in physiological traits associated with energy expenditure.

## Résumé

Pour les animaux sociaux, la vie en groupe est associée à plusieurs coûts et bénéfices. La présence de congénères peut limiter ou amplifier l'expression des comportements individuels. L'environnement social peut également affecter les réponses comportementales ultérieures d'un individu dans d'autres contextes, même lorsqu'il se retrouve seul. Or, les effets de l'environnement social sur l'expression des traits physiologiques individuels, comme les taux métaboliques, sont très peu connus. La vie en banc pourrait réduire les taux métaboliques des poissons. Cela dit, la compétition pour des composantes limitantes de l'habitat comme la présence de refuges pourrait influencer les taux métaboliques individuels. L'objectif de cette étude était de quantifier et de comparer les effets de l'environnement social et de la présence de refuges sur les taux métaboliques des ménés communs *Phoxinus phoxinus* estimés par respirométrie. Notre design expérimental consistait en une expérience sociale de trois semaines précédée et suivie par des expériences de respirométrie. Durant l'expérience sociale, les poissons étaient gardés en groupes de quatre ou huit poissons dans des aquariums qui contenaient un refuge (plante aquatique) ou non. Durant la respirométrie, les poissons étaient placés en isolation dans des chambres qui étaient couvertes par des plantes aquatiques pour la moitié de la durée des expériences. Ainsi, les taux métaboliques minimum de jour et de nuit, en présence ou en absence de refuge ont été estimés à chaque expérience, en plus des taux métaboliques standard (SMR) et maximum (MMR). Les taux métaboliques minimum de jour et de nuit estimés en présence de refuge étaient moins élevés que ceux estimés en absence de refuge, et ce, autant avant et après l'expérience sociale. Après l'expérience sociale, les SMR étaient plus élevés pour les poissons qui avaient été gardés en groupes de quatre que pour les poissons gardés en groupes de huit, alors qu'aucune différence n'a été observée pour les MMR. La présence de refuge dans les aquariums durant l'expérience sociale n'a pas influencé les taux métaboliques. Nos résultats démontrent que la taille des groupes peut influencer les dépenses énergétiques des individus, ce qui souligne l'importance de comprendre le rôle des dynamiques sociales sur les variations dans les traits métaboliques.

## Introduction

An animal social group is any set of socially interacting individuals that remain together in space and time (Krause and Ruxton 2002). Group living can provide a number of benefits, such as reduced predation risk, improved foraging, increased mate choice, and reduced energetic cost of movement or thermoregulation (Krause and Ruxton 2002; Evans et al. 2016; Jolles et al. 2020). Conversely, group living can be associated with increased conspicuousness or attack rates from predators, reduced individual growth if food resources are limited, and increased parasite or disease burden (Hoare et al. 2004; Altizer et al. 2003; Guénard et al. 2012). Social structures emerge in groups from variability in individual behavior and interactions among groupmates. Some behavioral responses are influenced by the number of groupmates present (Krause and Ruxton 2002). For example, group size has been negatively correlated with foraging in novel contexts (Day et al. 2001) but positively correlated with exploration (Ward 2012). Presence of conspecifics can restrict or enhance the expression of individual behavior through processes like conformity or facilitation (Ward 2012; Jolles et al. 2016; Ward and Webster 2016). Consequently, individuals may express a different suite of behaviors and different degrees of their full behavioral capacity while in group compared with when they are alone (Jolles et al. 2020). Further, there is some evidence that the recent social environment can affect behavioral responses in later contexts, even when individuals are alone (Jolles et al. 2016). This suggests that the social environment could modulate an individual's behavioral expression or capacity, yet the ways in which the phenotype of individual animals interacts with their social environment remain largely unknown, including how social dynamics affect individual physiological traits.

The interplay between the social environment and individual physiological traits may be especially complex due to the effects of social dynamics on individual stress, energy intake, and energy use (Webster and Ward 2011). For instance, standard metabolic rate (SMR), the minimum rate of energy use needed to sustain life at a given temperature in an ectotherm (Burton et al. 2011; Chabot et al. 2016), generally correlates with dominance, aggression, and tendency to take risks among individuals (Biro and Stamps 2010; Redpath et al. 2010; Metcalfe et al. 2016; Arnold et al. 2021). However, there is also evidence that individual stress can influence SMR over various temporal scales. In brown trout *Salmo trutta*, holding in pairs led to an increase in SMR of subordinate individuals, probably due to social stress, while SMR of dominant individuals did not change (Sloman et al. 2000). This is an example of how dominance can modulate relationships between metabolism and behavior (Killen et al. 2013), though whether such effects occur in larger or more complex social systems than dyads requires further investigation. There is evidence, however, that shoaling can reduce SMR in fish through “calming effects” (Nadler et al. 2016). Like SMR, maximum metabolic rate (MMR) and aerobic scope (AS; the difference between MMR and SMR) can correlate with dominance (Killen et al. 2014), boldness, or aggression (Redpath et al. 2010). However, to our knowledge, there is no evidence to date that social stress can influence MMR or AS (Killen Croft et al. 2016), despite their potential to constrain energetically costly behaviors and other aerobically fueled activities (Metcalfe et al. 2016). In any case, SMR and MMR are often positively correlated (Killen Glazier et al. 2016; Norin and Clark 2016; Auer et al. 2017) within and across species. As such, any effects of social dynamics on metabolic rates at rest may also affect aerobic capacity, or vice versa. The potential for social dynamics to influence either SMR or MMR could be reflected in AS, and thus influences the capacity to perform aerobically fueled activities. Yet, few studies have investigated how group living affects interactions between behavioral and physiological traits (Huang et al. 2020), aside from studies looking at effects of dominance in dyads (Sloman et al. 2000).

Habitat may further modulate interactions between individual traits and social dynamics (Jolles et al. 2020). Habitat conditions such as temperature or oxygen concentration influence metabolic rates, which in turn may affect performance among individuals within groups (Claireaux and Lefrançois 2007; Fry 1971; Huey 1991; Horodysky et al. 2015). Conversely, social stress can reduce tolerance to thermal stress (LeBlanc et al. 2011) and hypoxia (Thomas and Gilmour 2012). Other habitat conditions such as food and shelter availability may exert density-dependent influences on relationships between metabolism and behavior. A number of studies have revealed that SMR or resting metabolic rate estimated while in presence of shelter was reduced compared with when shelter was absent, probably due to decreased stress or reduction of alertness or vigilance when individuals are visually hidden (Fischer 2000; Finstad et al. 2004; Millidine et al. 2006; Norin et al. 2018; Chrétien et al. 2021). However, little is known about the effects of long-term shelter availability on individual metabolic rates and interactions with an animal's social environment. Increased competition for a limited resource, like availability of shelter, could strengthen social hierarchies and increase stress experienced by subordinates, and these effects could be greater in larger social groups. As such, group size and long-term shelter availability may have interacting effects that carry over and influence individual metabolic rates.

We investigated whether exposure to a given group size and shelter availability could influence metabolic rates of Eurasian minnow *Phoxinus phoxinus*, a small Cyprinid naturally living in social groups (Magurran 1986). We held fish in groups of four or eight, in tanks with or without plant shelter. The combination of group size and plant shelter availability in experimental tanks generated social treatments that differed in fish density and potential competition intensity for use of shelter. Respirometry trials were conducted before and after fish were housed for 3 weeks in these different social treatments, to measure metabolic rates (ṀO_2_). Furthermore, during respirometry trials, metabolic rates were estimated in two conditions that both lasted about half the duration of the trial: while respirometers were covered by plant shelter and while respirometers were not covered by such plant shelter. This design allowed us to get estimates of minimum daytime and nighttime metabolic rates (ṀO_2 min_) in presence or in absence of plant shelter, as the importance of being visually hidden by a shelter may vary with light intensity, as well as estimates of SMR, MMR, and AS. We hypothesized that the recent social environment, in the 3-week social treatment, would have metabolic costs that carry over, even when individuals are alone (Jolles et al. 2016), and be reflected in estimates of metabolic rates. Consequently, we predicted that presence of plant shelter during respirometry trials would lower daytime ṀO_2 min_, but that the magnitude of this effect would be smaller after the fish were held for 3 weeks in their social treatment (Killen et al. 2013). Given that minnows are social fish, we also predicted that SMR would vary with group size, due to the potential for social dynamics to modulate SMR (Sloman et al. 2000). We also predicted that fish held without access to plant shelter in their social treatment would have higher SMR, due to chronic effects of stress (Huey 1991). The potential for group size and plant shelter availability to influence MMR is unclear. On the one hand, MMR is generally thought to be less plastic than SMR (Norin and Metcalfe 2019), but on the other hand, SMR and MMR are thought to be positively correlated (Killen Glazier et al. 2016; Norin and Clark 2016). We nonetheless expected to see changes in AS due to predicted changes in SMR.

## Materials and methods

### Experimental animals

Juvenile Eurasian minnows (*P. phoxinus* Linnaeus) were captured in spring 2018 from River Kelvin (55.86667, −4.31667; Glasgow, United Kingdom) using dip nets. The sampling location was an artificial side channel along the River Kelvin where small minnows are trapped as they pass over a weir and are unable to return to the main river. Fish were transported to the nearby University of Glasgow aquarium facilities and held at 15°C in two large stock tanks (100 cm × 40 cm × 30 cm) each filled with 100–150 individuals (density = 833–1250 fish m^–3^) for 11 months before the study, which took place in April and May 2019. During this holding period, fish were fed *ad libitum* a combination of pellets and blood worms and were on a 12 h light:12 h dark photoperiod.

### Experimental design

**Fig. 1:**
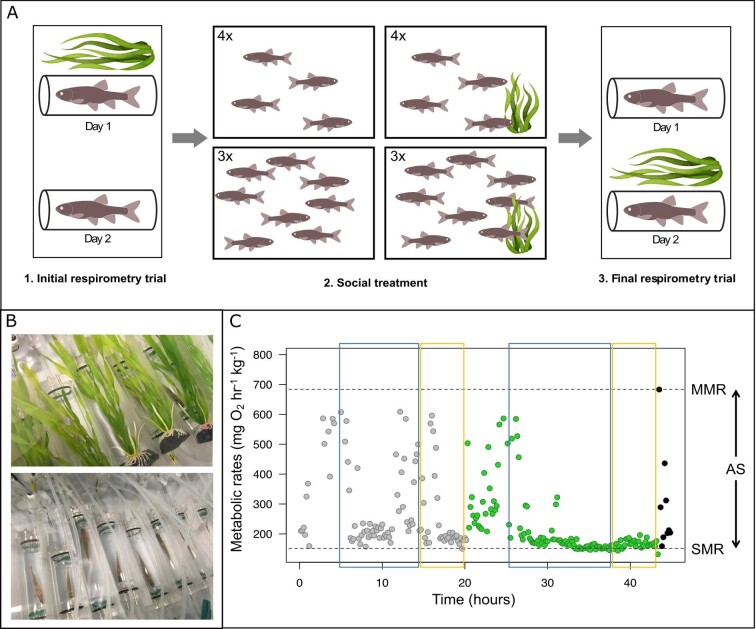
Experimental design of the study. **(A)** Each experiment consisted of an initial respirometry trial, a 3-week social treatment, and a final respirometry trial. (1) Initial respirometry trial: Fish oxygen uptake was measured for ∼45 h during which chambers were covered with artificial plant shelter for approximately half of the trial duration. (2) Social treatment: After the initial respirometry trial, fish were allotted in groups of four or eight fish and placed in experimental tanks containing artificial plant shelter or not, thus forming different social treatments. Fish stayed in their social treatment for 3 weeks. (3) Final respirometry trial: After the social treatment, fish oxygen uptake was measured again by respirometry, in chambers covered with artificial plant shelter for half of the trial duration. **(B)** Photograph of respirometry trial setup with fish in individual chambers covered with plant shelter (top) or not (bottom). **(C)** Experimental protocol to obtain ṀO2 data for the Eurasian minnow. The example shows a 48-h long respirometry trial that started with the condition “without plant shelter” (chamber not covered with plant shelter; gray points). The condition was changed to “with plant shelter” (green points) the next day at around noon. On the last day at noon, fish was removed from the respirometry chamber, chased, and immediately placed back into the chamber to obtain MMR (black points). Blue and yellow rectangles represent the range of data used for estimation of nighttime and daytime minimum ṀO2, respectively, with or without plant shelter. Top and bottom horizontal dotted lines show estimates of MMR and SMR.

Experiments were conducted on a total of 80 fish. Since the capacity of the respirometry setup was of 16 fish (each such group is hereafter referred to as a “batch”), five batches were subjected to respirometry before and after exposure to the social treatments (combination of group size and shelter availability). Each experiment consisted of an initial respirometry trial, a 3-week social treatment, and a final respirometry trial (Fig. 1). Before the onset of an experiment, a group of 16 minnows was haphazardly picked from the two stock tanks and isolated for 48 h in a rearing tank (40 cm × 40 cm × 30 cm). During that period, fish were fasted to ensure they were in a post-absorptive state before the respirometry trial.

Each respirometry trial was conducted to estimate fish metabolic rates in the presence or absence of artificial plant shelter. Fish were placed in individual glass chambers (∼100 mL) separated by opaque white dividers to prevent fish from seeing each other. Respirometry trials lasted ∼45 h during which chambers were covered with artificial plant shelter for approximately half of the trial duration (Fig. 1). The presence of an artificial plant over the chamber was randomly set to occur during the first or the second half of the initial respirometry trial (Fig. 1B), and order was reversed for the final respirometry trial. At the end of the initial respirometry trial, fish were weighed, measured, and injected with a unique combination of visible implant elastomer (Northwest Marine Technology, Anacortes, WA, USA) in the dorsal body surface to allow individual identification. The 16 fish within a given batch were afterward allotted in groups of four or eight fish (e.g., the first batch was allotted in four groups of four fish, the second batch was allotted in two groups of eight fish, and so on) and placed in experimental tanks (40 cm × 40 cm × 30 cm) containing artificial plant shelter or not, thus forming different social treatments. After the 3-week social treatment, the 16 fish were weighted and measured again, and the final respirometry trial was conducted. Testing the five batches, from the beginning of the initial respirometry trial with the first batch to the end of the final respirometry trial with the last batch, required 41 days.

Social treatments took place in 14 experimental tanks of identical dimensions (40 cm × 40 cm × 30 cm). In eight of these experimental tanks, the social treatment was defined by a group size of four fish (density = 83 fish m^–3^) either with, or without, artificial plant shelter (four experimental tanks each). In the remaining six experimental tanks, the social treatment was defined by a group size of eight fish (density = 166 fish m^–3^) either with, or without, artificial plant shelter (three experimental tanks each).

Fish were fed daily *ad libitum* a combination of pellets and blood worms, scattered throughout their experimental tank, during the 3-week social treatment to minimize potential effects of density on individual food intake and growth. Daily specific growth rate (SGR: in % day^–1^) during the 3-week social treatment was calculated for each individual using the following equation:

(1) \begin{equation*}{\rm{SGR}} = \frac{{\left[ {{\rm{log}}\left( {{\rm{Mf}}} \right){\rm{\ }} - {\rm{\ log}}\left( {{\rm{Mi}}} \right)} \right]}}{t}{\rm{\ \ }} \times 100,\end{equation*}

where *M*_f_ is the observed mass at the time of the final respirometry trial, *M*_i_ is the observed mass at the time of the initial respirometry trial, and *t* is the number of growth days. Over the 3-week social treatment, SGR was higher for fish held in groups of four (mean ± standard deviation: 0.64 ± 0.27% day^–1^, from −0.07 to 0.99% day^–1^, Fig. S1) than for fish held in groups of eight (0.50 ± 0.19% day^–1^, from 0.09 to 0.99% day^–1^), and this difference was significant (*P* = 0.004, *R*^2^_adj_ = 0.084). No relationship was found between SGR and metabolic rates measured at the final experiment (see Supplementary Information for details: Tables S1 and S2, Figs. S1 and S2).

### Respirometry trials

Metabolic rates were estimated using oxygen uptake rates (ṀO_2_: mg O_2_ h^–1^; Svendsen et al. 2016; Killen et al. 2021), determined via intermittent flow-through respirometry equipment and software (FireSting, PyroScience, Aachen, Germany). Water was continuously mixed through each chamber with a peristaltic pump and gas impermeable tubing. Automated flush pumps refreshed the chambers with UV-treated and oxygenated water for 2 min of every 7-min cycle. Dissolved oxygen concentrations were maintained above 80% air saturation at all times with air bubblers. Temperature was measured with a Pt100 temperature probe and maintained at 15°C with a TMP-REG instrument (Loligo Systems, Tjele, Denmark) by recirculation of water through a stainless coil in a cold bath.

Respirometry trials lasted ∼45 h (43.8–46.1 h), and chambers were covered with artificial plants for about half of their duration (∼21.5 ± 2 h; Fig. 1). Respirometry trials started mid-afternoon, and condition (with or without artificial plant shelter) was changed at around noon the next day (∼21 h after the onset of the respirometry trial). Approximately 43 h after the onset of the respirometry trial, fish were taken out of their chamber one by one for a 2-min chase protocol (Roche et al. 2013) and returned in their chamber for immediate measurement of ṀO_2_ to estimate their maximum metabolic rate MMR (Fig. 1C). Respirometry resumed for another hour, and fish were removed from the chambers and transferred to their original experimental tank. Background oxygen consumption in each empty chamber was recorded over three 7-min cycles at the start and end of each respirometry trial.

### Calculation of metabolic rates

**Fig. 2:**
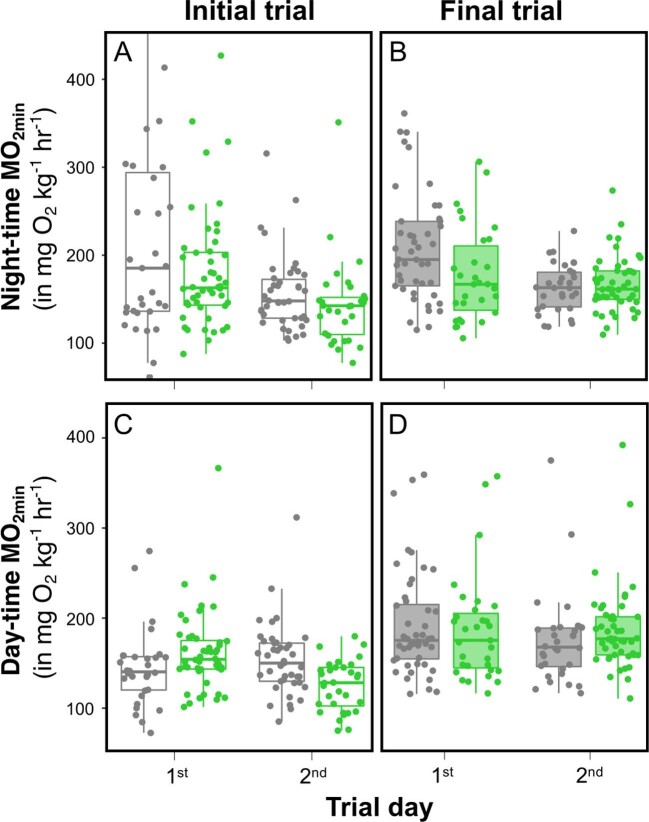
Observed nighttime **(A, B)** and daytime **(C, D)** metabolic rates in initial (clear) and final (shaded) respirometry trials. Gray and green dots represent estimates in absence or in presence of plant shelter, respectively. Middle thick line of the box plots corresponds to the median; lower and upper limits correspond to the first and third quartiles of the data; and whiskers extend to the range of the data.

Metabolic rates were calculated by multiplying the slopes of decline in oxygen concentration in the chamber during closed measurement cycles, excluding the first 30 s, by the volume of the chamber (corrected for the volume of fish, assuming a density of 1 kg L^–1^) using the package FishResp in R (R Foundation for Statistical Computing 2018; Morozov et al. 2019). Background oxygen consumption was subtracted from ṀO_2_ measurements, assuming a linear change between measures taken at the start and end of each trial. Daytime and nighttime minimum metabolic rates (ṀO_2min_; mg O_2_ kg^–1^ h^–1^) were calculated separately to account for the potentially different effect of the presence of shelter during daytime and nighttime. ṀO_2min_ were estimated using the 0.2 quantile of the ṀO_2_ data with the package fishMO2 in R (Chabot et al. 2016; Chabot 2016). The range of data used for the calculation of nighttime ṀO_2min_ started 5 h after fish were put in the chamber (at around 9:30 pm) or 5 h after the change in condition (presence of plant shelter or not; at around 6:30 pm), and ended in the morning at 7:00 am, moment at which lights were turned on. The range of data used for the calculation of daytime ṀO_2min_ started at 7:00 am and ended at the change in condition, or when fish were retrieved from the chamber for the chase protocol (Fig. 2). SMR (mg O_2_ kg^–1^ h^–1^) was set as the lowest estimate of daytime or nighttime ṀO_2min_ over a trial. MMR (mg O_2_ kg^–1^ h^–1^) was estimated as the highest rate of oxygen consumption over a 3-min rolling average regression within a measurement cycle following the chase protocol. AS (mg O_2_ kg^–1^ h^–1^) was calculated as the difference between MMR and SMR. All metabolic rates were adjusted to the mean body mass of the fish in our sample (mean ± standard deviation: 1.95 ± 0.57 g) using the slope *b* of the log–log relationship between ṀO_2_ and mass (Steffensen et al. 1994; Ultsch 1995).

(2) \begin{eqnarray*} {\rm{\dot{M}}}{{\rm{O}}_{2{\rm{adj}}}} &=& {\left( {{\rm{mean\ fish\ mass}}} \right)^{b - 1}}\! \times \! {\left( {{\rm{individual\ fish\ mass}}} \right)^{1 - b}}\nonumber\\ && \times \ {\rm{individual\ fish\ }}\dot{M}{O_2}\end{eqnarray*}

From each respirometry trial, two nighttime and daytime ṀO_2min_ estimates were calculated (one per trial day) per fish, as well as one SMR, MMR, and AS (Fig. 1C). This resulted in one dataset of 320 estimates of nighttime and daytime ṀO_2min_, and another dataset of 160 estimates of SMR, MMR, and AS, for 80 fish. Some data points were removed in the nighttime and daytime ṀO_2min_ dataset because slopes of decline in oxygen concentration in the chambers did not have sufficiently high *R*^2^ (>0.95), resulting in 306 estimates of nighttime ṀO_2min_ and 312 estimates of daytime ṀO_2min_, on 80 fish. In addition, two fish did not reach MMR during the initial respirometry trial; therefore, the final dataset comprises 158 estimates of MMR, and 158 estimates of AS, on 80 fish.

### Statistical analyses

All data are available from Zenodo (https://doi.org/10.5281/zenodo.4705121, Chrétien et al. 2021). All analyses were computed in R v. 3. 6. 0 (R Foundation for Statistical Computing 2018). Effects of presence of shelter on nighttime and daytime ṀO_2min_ measured during initial and final respirometry trials were tested using linear mixed effects models (LMM) with the package lme4 (Bates et al. 2014). Full models with nighttime or daytime ṀO_2min_ as a response variable included trial (initial or final), trial day (first or second), presence or absence of plant shelter covering respirometry chambers during the trial, fish body mass (g), and all two-way interactions as fixed effects. Trial day, fish body mass, and interaction terms were included in models in case they contributed to variation in estimates of metabolic rates, but dropped if nonsignificant and the models re-run. Models included fish ID and batch number (1 to 5) as random effects in a nested structure (batch number/fish ID). Model assumptions were met when response variables were log-transformed. For all models, assumptions of homoscedasticity, linearity and normality were confirmed by visual inspection of residual plots.

Effects of group size and shelter availability on SMR, MMR, and AS were tested with LMM using data from the initial and final respirometry trials, social treatment conditions (group size: four or eight fish; shelter availability: presence or absence of artificial plant in experimental tank), fish body mass, and all interactions as fixed effects. The SMR model included fish ID and batch number as random effects in a nested structure (batch number/fish ID). The MMR and AS models included only fish ID as random effect. Model assumptions were confirmed by visual inspection of residual plots.

Effect sizes (in %) were calculated using estimated marginal means from final models obtained with the package emmeans (Lenth and Hervé 2015). Marginal *R*^2^ (*R*^2^_m_: % of variance explained by fixed effects) and conditional *R*^2^ (*R*^2^_c_: % of variance explained by fixed and random effects) were calculated from the models fitted through restricted maximum likelihood analysis (Bolker et al. 2009; Harrison et al. 2018). The difference between *R*^2^_c_ and *R*^2^_m_ for each model represents variability due to the random effects (Nakagawa and Schielzeth 2013).

## Results

### Presence of shelter during respirometry trials

Respirometry timing (initial or final), trial day, and plant shelter (presence or absence during respirometry) had significant effects on nighttime ṀO_2min_ (*P* = 0.002, *P* < 0.001, and *P* = 0.002, respectively; Table 1). Nighttime ṀO_2min_ estimates were on average 8.7% higher during the final respirometry trial compared with that of the initial one. They were also 16.2% lower on the second day of trial compared with the first day, and 7.9% lower in the presence of plant shelter (Fig. 2A and B) compared with when plant shelter was absent. Daytime ṀO_2min_ was influenced by respirometry timing (*P* < 0.001; Table 1). Daytime ṀO_2min_ was on average 26.9% higher at the final respirometry trial (Fig. 2C and D). There was an interaction between trial day and plant shelter on daytime ṀO_2min_ (*P* = 0.044): in the presence of plant shelter, daytime ṀO_2min_ rates measured on the second day were 10.0% lower than those of the first day.

**Table 1:** Results of linear mixed models relating night-time and day-time minimum metabolic rates (ṀO_2min_) of Eurasian minnows to respirometry trial (initial or final), trial day, and presence or absence of plant shelter. *R*^2^_m_ is the marginal *R*^2^ (% of variance explained by the fixed effects) and *R*^2^_c_ is the conditional *R*^2^ (% of variance explained by the fixed and the random effects).

Response variable	Effect	χ^2^	*P*-value	*R* ^2^_m_	*R* ^2^_c_
log Nighttime ṀO_2min_	Trial	9.313	0.002	11.5	42.6
	Day	42.501	<0.001		
	Plant shelter	9.229	0.002		
log Daytime ṀO_2min_	Trial	111.905	<0.001	16.7	56.2
	Day	7.591	0.006		
	Plant shelter	0.052	0.819		
	Day*plant shelter	4.051	0.044		

### Social treatments and metabolic rates

**Fig. 3:**
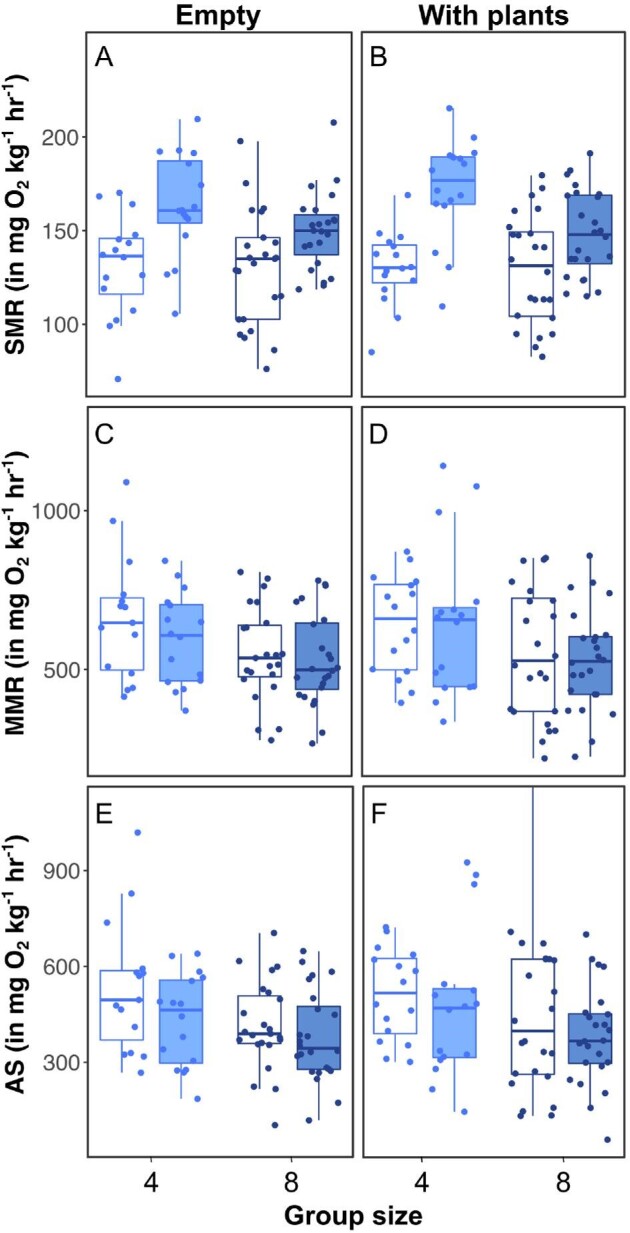
Observed SMR **(A, B)**, MMR **(C, D)**, and AS **(E, F)** of Eurasian minnow. Light blue and dark blue boxes and points represent estimates for fish held in groups of four and eight, respectively. Clear and shaded boxes represent initial and final respirometry trials, respectively. Panels A, C, and E refer to experimental tanks without plant shelter, while panels B, D, and F refer to experimental tanks containing plant shelter. Middle thick line of the box plots corresponds to the median; lower and upper limits correspond to the first and third quartiles of the data; and whiskers extend to the range of the data.

There was an interacting effect of trial and group size (*P* = 0.006; Table 2) on SMR. Estimates of SMR were 28% higher at the final respirometry trial compared with the initial one for fish held in groups of four, while SMR increased 13% between the two trials for fish held in groups of eight (Fig. 3A and B). Plant shelter availability in experimental tanks did not influence SMR. MMR did not change between the initial and final respirometry trials (*P* = 0.254). Fish held in groups of four had, however, higher MMR than fish held in groups of eight (*P* = 0.005; Fig. 3C and D). Finally, there was an overall reduction in AS after the 3-week social treatment (*P* = 0.029; Table 2). Group size also negatively influenced AS (*P* = 0.008; Fig. 3E and F). Plant shelter availability in experiment tanks did not influence MMR or AS (Table 2).

**Table 2:** Results of linear mixed model relating metabolic rates of Eurasian minnows to the moment of the respirometry trials, group size, and shelter availability. Fish ID and batch number were included in the SMR model as random effects. Only fish ID was included as a random effect for MMR and AS models. *R*^2^_m_ is the marginal *R*^2^ (% of variance explained by the fixed effects) and *R*^2^_c_ is the conditional *R*^2^ (% of variance explained by the fixed and the random effects).

Response variable	Effect	χ^2^	*P*-value	*R* ^2^_m_	*R* ^2^_c_
SMR	Trial	54.646	<0.001	19.6	54.7
	Group size	0.469	0.494		
	Shelter availability	0.009	0.925		
	Trial*group size	7.567	0.006		
MMR	Trial	1.302	0.254	6.3	24.6
	Group size	7.795	0.005		
	Shelter availability	0.226	0.636		
AS	Trial	4.740	0.029	7.3	24.2
	Group size	6.887	0.008		
	Shelter availability	0.254	0.614		

## Discussion

The main goal of this study was to assess whether being in a smaller or larger group of conspecifics and having plant shelter available or not could modulate expression of metabolic traits. Both before and after fish were exposed to different social treatments, minimum metabolic rates estimated in presence of shelter were lower than those estimated in absence of shelter. This indicates that plant shelter availability during respirometry trials has a consistent and robust lowering effect on estimates of minimum metabolic rates in Eurasian minnow, as the recent social treatment did not mask this effect. We did, however, observe an overall increase in estimates of SMR between the initial and final respirometry trial, with the increase in SMR throughout the study being two-fold higher for fish held in groups of four as compared with that of fish held in groups of eight. Availability of plant shelter in experimental tanks during the social treatments did not affect metabolic rates. Whether such effects would be similar if fish could see their conspecifics during the respirometry trials remains to be tested. Nonetheless, our results suggest that recent social group size can have metabolic effects that carry over, even when fish are at rest and in isolation, such as during respirometry trials. This means that group size could have a modulating effect on levels of baseline metabolism, which could in turn have implications on an animal's energy budget, including growth, reproductive investment, and overall performance capacity. In the current study, the presence of more groupmates in the social treatment was associated with lower metabolic rate, suggesting that a reduction in energy demand may be an additional benefit of living in larger social groups.

### Presence of shelter during respirometry trials

Presence of plant shelter during respirometry trials lowered estimates of metabolic rates both before and after exposure to the social treatments. Presence of shelter during respirometry trials has been associated with lower metabolic rates in some species (Fischer 2000; Finstad et al. 2004; Millidine et al. 2006; Norin et al. 2018) but not in others (Fischer 2000; Kegler et al. 2013), or to mixed results (Chrétien et al. 2021). Using shelter can reduce the occurrence of otherwise energetically demanding activities, such as those associated with maintaining vigilance against predators (Lind and Cresswell 2005; Millidine et al. 2006). It was surprising that the effect of shelter was stronger for nighttime than for daytime ṀO_2min_, assuming the main reason for sheltering is to remain visually hidden. This pattern was nonetheless observed in another study, where an effect of shelter presence was observed during the night but not during the day (Norin et al. 2018). It is possible that fish showed higher levels of spontaneous activity during daytime that might mask any effect of the shelter on ṀO_2min_, although no consistent relationship has been observed between activity and light intensity in our study species (Jones 1956). Another potential explanation is that fish had time to acclimate to the presence of shelter before lights were turned off for the night, and therefore anticipated that they could be sheltered at night. In the laboratory, lights were turned off at 7:00 pm, so about 3–6 h after plant cover was placed over the chambers (depending if this condition occurred on the first or the second day of the respirometry trial). We predicted that the magnitude of the effect of shelter on metabolic rates would be smaller after the 3-week social treatment. This trend was not observed, suggesting that individuals did not adjust their metabolic response to immediate shelter presence, regardless of the group size or level of shelter availability they received during the social treatment. This indicates that shelter availability has a consistent and robust lowering effect on resting metabolic rates in Eurasian minnow and likely other species with similar social systems and patterns of habitat use.

### Social treatments and metabolic rates

There was an overall increase in estimates of SMR throughout the study. Importantly, group size affected the strength of the increase: fish held in groups of four showed a two-fold higher increase in estimated SMR than fish held in groups of eight. Although Eurasian minnows can be found in small groups in the wild (similar to that used during the social treatments of the current study), they can also form much larger shoals (Magurran 1986; S. S. Killen, University of Glasgow, personal observation). While it is therefore possible that the larger group size has a buffering effect on individual metabolic costs, through improved security or reduced individual vigilance (Nadler et al. 2016; Culbert et al. 2019), caution must be exercised when extrapolating the trends observed here to groups with hundreds of fish in nature. We cannot rule out that conditions may have been more favorable for growth in tanks with groups of four fish, even if food was not a limited resource in any social treatment. However, there was no relationship between final SMR and SGR, nor was there an interaction between SGR and social treatment conditions (Tables S1 and S2; Figs. S1 and S2), suggesting other mechanisms are more likely to explain the differences observed. Since all experimental tanks were of the same size, densities varied between group size (density_4fish_ = 83 fish m^–3^; density_8fish_ = 166 fish m^–3^). Therefore, the differences observed could be either due to differences in group sizes or densities. For instance, fish in groups of four potentially had more volume available for individual exploration and an increased need for individual vigilance, potentially increasing the cognitive load and associated metabolic costs that may carry over, even when the fish are at rest, during respirometry for estimates of SMR (Moss et al. 1998). Prolonged changes in locomotor activity level due to social interaction or vigilance may induce changes in muscle enzyme levels and mitochondria density, and thus affect fish minimum energy demand (Killen Glazier et al. 2016).

Intensity of competition and strength of hierarchy structures could also vary with group sizes. For example, Pottinger and Pickering (1992) observed that social hierarchies emerged in rainbow trout *Oncorhynchus mykiss* held for 6 weeks in pairs or in groups of 5, but not in groups of 10 fish. An increase in aggressive behavior such as pecking incurs increased activity and metabolic costs (Marchand and Boisclair 1998). With increasing group size, competition for limited resources like shelter may increase but dominance hierarchy tends to weaken, as the cost of interacting with multiple individuals may become too high (Sloman and Armstrong 2002).

We did not observe any statistically significant difference in SMR for fish held in experimental tanks containing plant shelter or not. It is possible that plant shelter in the experimental tanks were considered as a limited resource that stimulated competition, especially for the 8-fish groups. In experimental tanks with plant shelter available, only one artificial plant was provided, meaning that for the 8-fish groups, there was relatively less per capita shelter available than for the 4-fish groups, which could have enhanced social stress. Sustained stress in social groups with stronger dominance hierarchies could also carry over and limit our ability to effectively estimate SMR (Sloman et al. 2000; Killen et al. 2014; Metcalfe et al. 2016). Additional research on the effects of social dynamics on fish cognitive abilities or stress indicators could shed light on the mechanisms underlying the results observed here.

We did not expect group size to affect metabolic rates in the initial respirometry trial as fish were all held in the same high-density stock tank beforehand. Yet, fish held in groups of four had significantly higher MMR and AS than fish held in groups of eight before the 3-week social treatment (Table 2). However, this result seems to be driven by a single batch of fish. Groups of four fish were created from the first and the fourth batches while groups of eight fish were created from batches two, three, and five. Only the first batch of 16 fish subjected to our experiment reached overall higher MMR (and AS) than the other batches at the initial respirometry trial (Fig. S3). One hypothesis that might explain the observed result is that the first batch may have been composed of individuals with higher susceptibility to capture, a trait that can be associated with higher metabolic rates (Redpath et al. 2010). While this pattern could be interesting to investigate in other studies, we can only interpret it here as a measurement artefact and cannot link this result to the social treatments. We conducted respirometry trials before and after the social treatments first to account for initial differences in metabolic rates, and second to quantify the relative change in metabolic rates after the social treatments. Regardless of initial differences in metabolic rates, that may be driven by the first batch of fish subjected to our experiment, results from models show there was no significant difference in MMR between trials, suggesting no relative change in MMR throughout the experiment. In addition to statistically controlling for effects from initial differences in metabolic rates by including “trial” and its interaction with other effects in full models, we also included “batch number” as a potential random effect in all our models to account for higher similarities in fish from the same batch compared with other fish. Batch number was retained in a nested structure with fish ID for nighttime ṀO_2min_, daytime ṀO_2min_ and SMR models. It was not, however, kept in models on MMR or AS, because its inclusion resulted in singular fits (Matuschek et al. 2017): no variance was associated with the random effect “batch number.” In any case, models using either “fish ID” or “batch number/fishID” as a random component generated similar results (Table S3). We consider that our statistical approach was robust to control for initial differences in metabolic rates, and to higher similarities in fish from the same batch compared with other fish. Our results, however, illustrate the importance of accounting for unforeseen or unforeseeable initial differences in metabolic rates when designing experiments. For instance, high susceptibility to capture is a trait that can correlate with MMR (Redpath et al. 2010), and might explain the pattern we observed when comparing MMR of the first batch of fish “captured” in the stock tank to MMR of the subsequent ones. One way to overcome this issue would have been to systematically allocate the 16 fish from each batch to two groups of four and one group of eight, instead of randomly assigning all fish from a given batch to a unique group size. Another way would be to avoid using the first batch of fish “captured” in a stock tank for experiments. Testing approaches to control for initial differences in metabolic rates would be useful to improve experimental designs, and should be the focus of further research.

The effect of the social environment on trait plasticity has been widely studied in behavioral ecology, but generally overlooked in comparative physiology (Gilmour et al. 2005). Yet, the social environment can influence individual stress levels and in turn affect the ability to tolerate additional stressors, like thermal stress (Leblanc et al. 2011) and hypoxia (Thomas and Gilmour 2012), especially in subordinate individuals. Conversely, group living has been associated with a reduction in overall metabolic demand, likely through a reduced need for individual vigilance (Roberts 1996). Similarly, shoaling has been suggested to have a “calming effect” and to reduce metabolic rates of social fish species, through conspecific visual and olfactory cues (Nadler et al. 2016). There is a need to consider how the social environment may affect physiological responses as, on the one hand, social dynamics may increase individual stress, but on the other hand, living in social groups may buffer physiological responses to some stressors (Culbert et al. 2019). We observed that group size could influence SMR in Eurasian minnows, which can be attributable to increased social stress at lower densities for these social fish. The study of interactions among individuals, dominance ranks, and robustness of dominance structure in the different social treatments could shed light on the results obtained here. It is possible that increased group size and habitat complexity induces metabolic plasticity, which suggests that selection on energy expenditure in animals with strong social systems may be less likely to result in genetic change. Our results highlight the importance of understanding the role of social dynamics on variations in individual metabolic traits and thus on the physiological consequences of habitat selection (Huey 1991).

## Supplementary Material

obab032_Supplemental_File

## Data Availability

The data underlying this article are available in Zenodo repository at https://doi.org/10.5281/zenodo.4705121, and can be accessed with doi: 10.5281/zenodo.4705121.
