# Supplementary material for: Social Group Size and Shelter Availability Influence Individual Metabolic Traits in a Social Fish
Source: Integr Org Biol. 2021 Nov 27;3(1):obab032. doi: 10.1093/iob/obab032 (PMC8633746; doi:10.1093/iob/obab032)
Supplement: obab032_Supplemental_File [file obab032_supplemental_file.docx]

**Social group size and shelter availability influence individual metabolic traits in a social fish**

Supporting Information

**Specific growth rate**

Fish were fed *ad libitum* a combination of pellets and blood worms in their experimental holding tank during the 3-week social treatment to minimize potential effects of density on individual food intake and growth. We compared daily specific growth rate (SGR: in % day^-1^) across social treatments and observed that fish held in groups of eight had on average lower SGR (Table S1), but that SGR was overall very variable among individuals (Figure S2).

Further, we tested if SGR could influence metabolic rates. It was not possible to include SGR in the models presented in the results of the manuscript as those models included more than one estimate of metabolic rates per individual (initial vs final respirometry trial), while we have a single SGR per individual. Therefore, we tested if SGR and its interaction with the social treatment could influence metabolic rates estimated at the final respirometry trial. There was no effect of SGR on any metabolic rate (Table S2, Figure S3). In addition, there was no interaction between SGR and group size or shelter availability.

Table S1: Results of linear model relating specific growth rate (SGR) of Eurasian minnows to the social treatment (group size and shelter availability).

| Response variable | Effect | Estimate  ± standard error | *F* | p-value | R^2^_adj_ |
| --- | --- | --- | --- | --- | --- |
| SGR | Group size | -0.152 ± 0.051 | 9.011 | 0.004 | 8.4 |
|  | Shelter availability | 0.025 ± 0.050 | 0.248 | 0.620 |  |

Table S2: Results of linear model relating final standard metabolic rate (SMR), maximum metabolic rate (MMR) and aerobic scope (AS) of Eurasian minnows to the specific growth rate (SGR), the social treatment (group size and shelter availability), and their interaction*.

| Response variable | Effect | *F* | p-value |
| --- | --- | --- | --- |
| Final SMR | SGR | 2.036 | 0.158 |
|  | Group size | 12.232 | <0.001 |
|  | Shelter availability | 0.190 | 0.664 |
| Final MMR | SGR | 0.028 | 0.868 |
|  | Group size | 4.983 | 0.029 |
|  | Shelter availability | 0.285 | 0.595 |
| Final AS | SGR | 0.001 | 0.972 |
|  | Group size | 3.182 | 0.078 |
|  | Shelter availability | 0.235 | 0.629 |

*All interactions were non-significant and were therefore removed from models.

Figure S1: Boxplot of specific growth rate (SGR; in % day^-1^) of fish in each social treatment consisting in a combination of group size (left: four fish; right: eight fish) and shelter availability (no plant shelter in blue; with plant shelter in turquoise). Middle thick line of the boxplots corresponds to the median, lower and upper hinges correspond to the first and third quartiles of the data, and whiskers extend to the range of the data. Each fish SGR is overlayed on the boxplot (black dots).

Figure S2: Observed SMR, MMR, and AS of Eurasian minnow (in mg O_2_ kg^-1^ hr^-1^) during the initial (top row) and final (bottom row) respirometry trial in relation to specific growth rate (SGR; in % day^-1^).

**MMR and AS**

Table S3: Results of linear mixed model relating maximum metabolic rate (MMR) and aerobic scope (AS) of Eurasian minnows to the moment of the respirometry trials and the social treatment (group size and shelter availability). Fish ID and batch number were included in the models as a random effect in a nested structure (batch number/Fish ID). R^2^_m_ is the marginal R^2^ (percentage of variance explained by the fixed effects) and R^2^_c_ is the conditional R^2^ (percentage of variance explained by the fixed and the random effects).

| Response variable | Effect | χ^2^ | p-value | R^2^m | R^2^c |
| --- | --- | --- | --- | --- | --- |
| MMR | Trial | 1.312 | 0.252 | 6.3 | 24.7 |
|  | Group size | 6.717 | 0.010 |  |  |
|  | Shelter availability | 0.224 | 0.636 |  |  |
| AS | Trial | 4.813 | 0.028 | 7.5 | 23.2 |
|  | Group size | 7.159 | 0.007 |  |  |
|  | Shelter availability | 0.262 | 0.609 |  |  |

Figure S3: Maximum metabolic rates (MMR) per batch of fish captured together from the stock tank. Left panel shows rates measured in the initial respirometry trial, right panel shows rates measured at the final respirometry trial. Middle thick line of the boxplots corresponds to the median, lower and upper hinges correspond to the first and third quartiles of the data, and whiskers extend to the range of the data. Each fish MMR is overlayed on the boxplot (black dots).
